# DUNEuro—A software toolbox for forward modeling in bioelectromagnetism

**DOI:** 10.1371/journal.pone.0252431

**Published:** 2021-06-04

**Authors:** Sophie Schrader, Andreas Westhoff, Maria Carla Piastra, Tuuli Miinalainen, Sampsa Pursiainen, Johannes Vorwerk, Heinrich Brinck, Carsten H. Wolters, Christian Engwer

**Affiliations:** 1 Institute for Biomagnetism and Biosignalanalysis, University of Münster, Munster, Germany; 2 Applied Mathematics: Institute for Analysis and Numerics, University of Münster, Munster, Germany; 3 Institute for Bioinformatics and Chemoinformatics, Westphalian University of Applied Sciences, Gelsenkirchen, Germany; 4 Radboud University Nijmegen Medical Centre, Donders Institute for Brain, Cognition and Behaviour, Nijmegen, The Netherlands; 5 Computing Sciences, Tampere University, Tampere, Finland; 6 Department of Applied Physics, University of Eastern Finland, Kuopio, Finland; 7 Institute of Electrical and Biomedical Engineering, UMIT - Private University for Health Sciences, Medical Informatics and Technology, Hall in Tyrol, Austria; 8 Otto Creutzfeldt Center for Cognitive and Behavioral Neuroscience, University of Münster, Munster, Germany; Cook Children’s Medical Center, UNITED STATES

## Abstract

Accurate and efficient source analysis in electro- and magnetoencephalography using sophisticated realistic head geometries requires advanced numerical approaches. This paper presents DUNEuro, a free and open-source C++ software toolbox for the numerical computation of forward solutions in bioelectromagnetism. Building upon the DUNE framework, it provides implementations of modern fitted and unfitted finite element methods to efficiently solve the forward problems of electro- and magnetoencephalography. The user can choose between a variety of different source models that are implemented. The software’s aim is to provide interfaces that are extendable and easy-to-use. In order to enable a closer integration into existing analysis pipelines, interfaces to Python and MATLAB are provided. The practical use is demonstrated by a source analysis example of somatosensory evoked potentials using a realistic six-compartment head model. Detailed installation instructions and example scripts using spherical and realistic head models are appended.

## Introduction

We present *DUNEuro*, an open-source software toolbox for the numerical computation of forward solutions in bioelectromagnetism. Its main focus is to provide an extendable and easy-to-use framework for using various finite element method (FEM) implementations for different neuroscientific applications, such as the electroencephalography (EEG) or magnetoencephalography (MEG) forward problems [1–3].

Forward solutions are a crucial component for accurate EEG and MEG source analysis results. Various methods for these computations have been suggested besides the finite element method (FEM), e.g., the boundary element method (BEM) [4–6], the finite volume method (FVM) [7] and the finite difference method (FDM) [8–10]. Our software focuses on FEM discretizations, which are able to cope with anisotropic tissue as well as complex geometries and have been widely used in EEG/MEG forward and inverse analysis [11–14]. Besides the standard Lagrangian Continuous Galerkin (CG-)FEM, modern FEM variants include the Discontinuous Galerkin method (DG-FEM) and the Unfitted Discontinuous Galerkin method (UDG-FEM). DG-FEM shows higher accuracies in low-resolution scenarios in which a thin skull layer may produce physically inaccurate discretizations [15, 16]. UDG-FEM does not require the generation of volumetric meshes but relies on an implicit description of the tissue layers via level-sets [17, 18]. In addition to FEM discretizations, there are various approaches to numerically treat the singular source term in the EEG forward problem. The approaches currently supported by DUNEuro include the St. Venant, the partial integration, the Whitney and the subtraction source models [15–20]. While the above-mentioned publications use DUNEuro for numerical calculations, their focus lies on mathematical derivations, studies on numerical convergence to the (quasi-)analytical solutions in spherical head volume conductor models as well as comparisons to existing methods such as CG-FEM. Based on these findings, this paper is intended to focus on the underlying software-related aspects in order to make the presented methods more accessible to the neuroscience community.

The goal of DUNEuro is to provide an open-source software toolbox offering sophisticated FEM discretizations as well as various source modeling approaches with convenient features for users as well as developers. From a user perspective, it is important that the toolbox is accessible and easy to use. Therefore, the interface of DUNEuro is designed in a way that different methods, such as discretization schemes, can be easily exchanged by passing different parameters as input. Additionally, DUNEuro provides interfaces to Python and MATLAB so that the user is not exposed to the high complexity of a C++ finite element code and the forward solutions can easily be embedded in an already existing analysis pipeline. As input, DUNEuro requires a head volume conductor model, sensor characteristics (sensor locations and for MEG additionally the direction in which the magnetic field is measured), as well as a set of dipole locations and moments, i.e., the source space. As output, DUNEuro provides accurate forward solutions for different applications like source analysis or the optimization of sensor configurations in brain stimulation. From a developer point of view, the DUNEuro toolbox is easily extendable. Different finite element methods share several subcomponents, such as the representation of the computational domain or the solver of the linear system. To facilitate the implementation of different discretization schemes or the extension of already existing approaches, DUNEuro is therefore designed to bundle different implementations and enable code reuse. Although DUNEuro currently supports only EEG and MEG forward solutions, transcranial electric and magnetic stimulations (TES/TMS), which are mathematically closely related due to Helmholtz reciprocity [21–23], are currently being integrated. Additionally, our toolbox makes use of components and benefits from existing maintenance and testing infrastructure as a module of the large DUNE framework, as described in more detail below.

In order to perform analysis of EEG or MEG data, different open-source software packages offer tools to implement a complete processing pipeline such as the MATLAB-based toolboxes FieldTrip [24], Brainstorm [25] and Zeffiro [26], the Python-based toolboxes MNE-Python [27], FEMfuns [28] or the C++ code MNE [29]. The toolbox SimBio (https://www.mrt.uni-jena.de/simbio) offers a broad range for forward and inverse methods for EEG and MEG analysis. Recently, the EEG FEM forward modeling using the St. Venant source model has been integrated into FieldTrip [30]. With respect to the finite element method, only first-order Lagrangian elements are fully implemented in SimBio. Due to its structure, extending the code to support different discretization schemes would be error-prone and time-consuming. In addition, there are well-tested and established general purpose software toolboxes for finite element computations.

One existing library for finite element computations is DUNE, the *Distributed and Unified Numerics Environment* (http://www.dune-project.org). More precisely, it is a general purpose open-source C++ library for solving partial differential equations using mesh-based methods [31]. It is extendable by offering a modular structure and providing abstract interfaces and separation between data structures and algorithms. Due to the modular structure, a user of DUNE only has to use those modules that are needed for a specific computation. At the core of the DUNE library is an abstract definition of a grid interface [32, 33]. Using the abstract interface allows writing reusable code that is independent of the concrete implementation of the grid or the type of the grids elements. This way, the identical code can be used in multiple spatial dimensions and for tetrahedral, hexahedral or other element types. DUNEuro builds upon several existing DUNE modules such as the dune-uggrid module [34] and the dune-pdelab module [35].

Currently, the DUNEuro toolbox is developed and used on Linux operating systems. The software is made available under the GPL open-source license and can be downloaded from the GitLab repository (https://gitlab.dune-project.org/duneuro), which is also linked from the DUNEuro homepage (http://www.duneuro.org), see also S1 Appendix for detailed installation instructions. The GitLab repository also contains a Wiki (https://gitlab.dune-project.org/duneuro/duneuro/-/wikis) with detailed documentation of the user interface including a description of parameters for using different FEM and source models.

In the following, we first give a short summary on the background of solving the EEG and MEG forward problems with finite element methods. This is followed by a general description of the structure and design of the toolbox, its use of existing frameworks for solving partial differential equations and its main concepts of different interfaces used within the library. Afterwards, we will describe a method for localizing elements within a tessellation based on a global coordinate. The interaction of a user with DUNEuro is done through bindings with a scripting language, which will be presented in the following section. Subsequently, we demonstrate how DUNEuro can be embedded in a complete analysis pipeline by calculating forward solutions which are then used for source analysis on EEG data obtained from a somatosensory experiment. Finally, a short summary and outlook is given. For detailed installation instructions of the DUNEuro toolbox, see S1 Appendix. In S2 Appendix, several example data sets and scripts in Python and MATLAB are provided and explained in detail.

## Background

In this section we present the background for solving the EEG and MEG forward problems with finite element methods.

### The EEG forward problem

The aim of the EEG forward problem is to compute the electric potential *u* in the head domain $\Omega \subset R^{3}$ [1–3]. It can be formulated using Poisson’s equation:

$$\begin{array}{ccc} \nabla \cdot \sigma \nabla u & =\nabla \cdot j^{p} & \text{in}\;\Omega \\ \langle \sigma \nabla u,\;n\rangle & =0 & \text{on}\;\partial \Omega , \end{array}$$ (1)

where $\sigma :\Omega \mapsto R^{3\times 3}$ denotes the symmetric and positive definite conductivity tensor, *j*^*p*^ denotes the primary current density and *n* denotes the unit outer normal on the head surface ∂Ω. The current source is usually modeled as a current dipole at a position *x*_dp_ ∈ Ω with the dipole moment $q\in R^{3}$. With the use of the delta distribution $\delta_{x_{\text{dp}}}$ centered at *x*_dp_, the current dipole leads to the source term

$$\begin{array}{c} \nabla \cdot j^{p}=q\cdot \nabla \delta_{x_{\text{dp}}}. \end{array}$$

### Finite element methods for the EEG forward problem

Several finite element methods have been proposed to solve the EEG forward problem. A common basis for these methods is the tesselation of the head domain Ω. The domain is partitioned into a set of elements of simple shape, such as tetrahedrons or hexahedrons. Depending on the concrete method, certain regularity assumptions are imposed on the tesselation, such as not containing hanging nodes. For details on the precise definition of a tesselation we refer to [36]. In the following, we will denote a tesselation of Ω by $T_{h}\left(\Omega \right)$. Here, h∈R denotes the maximal diameter of a mesh element.

Instead of searching for a solution in the infinite-dimensional function space in which the EEG forward problem 1 is posed, the finite element method relies on the construction of a finite-dimensional subspace in which the problem is discretized. Therefore, the potential *u* in 1 is approximated by a discrete representation denoted *u*_*h*_, which is defined on $T_{h}\left(\Omega \right)$. One main difference between several finite element methods is the choice of this discrete representation *u*_*h*_ for the potential and the formulation of the discrete representation of Poisson’s equation. We will not present the mathematical rigorous definition of the different methods but refer to the respective publications that introduced the methods for solving the EEG forward problem. The conforming finite element method using Lagrangian elements represents the potential as a continuous, piecewise polynomial function [12–14, 37–45]. For the discretization of the equation, the classical weak formulation is used directly. In contrast, the discontinuous Galerkin method does not enforce the continuity of the potential in its function definition but instead incorporates the continuity weakly through the use of a modified weak formulation [15]. Using this approach, the solution gains continuity of fluxes on the discrete level. Another approach, although currently not yet supported in DUNEuro, is the mixed finite element method (Mixed-FEM). To obtain the Mixed-FEM formulation, additional unknown variables are introduced, in the case of the EEG forward problem the electric current *j*. Thereby, the second-order Poisson Eq 1 is transformed into a system of first-order equations with both the electric potential *u* and the electric current *j* as unknowns. As a consequence of this discretization, the Mixed-FEM is current preserving, in contrast to the CG-FEM. A theoretical derivation of the Mixed-FEM approach for EEG forward simulations is provided in [46].

The finite element methods described above have in common that they use a tesselation which resolves the computational domain Ω. Recently, two finite element methods have been introduced for solving the EEG forward problem that instead use a tesselation of an auxiliary domain $\hat{\Omega }$ which is independent of Ω. The conformity of the solution to the domain Ω is incorporated weakly by modifying the discrete weak formulation. The CutFEM method uses a function representation that is continuous on each isotropically homogenized tissue compartment [18, 47]. The unfitted discontinuous Galerkin method additionally transfers the continuity constraint within each subdomain to the weak formulation [17, 48].

Due to the difference in representing the discrete function and the different properties of these representations, different strategies for discretizing the dipolar source term have to be taken into account. In general, it is unclear how to evaluate the derivative of the delta distribution in 1. Several different approaches for the various finite element methods have been proposed in the literature to handle this singularity. For the conforming finite element methods, the partial integration approach [14, 49], the St. Venant approach [11, 38, 50, 51], the full and projected subtraction approach [3, 40, 41, 52] and the Whitney approach [20, 39, 53–55] have been introduced. Similar approaches have been presented for the discontinuous Galerkin method, however their exact formulations differ, due to the different discretization approach. These approaches are the partial integration approach [56], the St. Venant approach [18] and the full and localized subtraction approach [15, 18]. For the unfitted finite element methods both the partial integration approach and different variants of the St. Venant approach have been adopted [17, 18].

A list of source models currently supported by DUNEuro for different FEM discretizations is provided in Table 1.

**Table 1:** Overview of source models currently supported for EEG/MEG by DUNEuro for different FEM discretization schemes.

		Source models			
		Partial integration [54]	St. Venant [54]	Subtraction [3, 52]	Whitney ^1^ [20]
**FEM**	**CG** [11]	EEG/MEG	EEG/MEG	EEG/MEG	EEG/MEG
	**DG** [15, 16]	EEG/MEG^2^	-	EEG/MEG^2^	-
	**UDG** [17]	EEG	-	EEG	-

^**1**^ The Whitney source model is currently only implemented for tetrahedral meshes.

^**2**^ In the MEG implementation, the numerical flux for the secondary magnetic field for DG-FEM is currently only implemented for hexahedral meshes.

In general, source models can be divided into direct and indirect approaches. Direct source models include the partial integration, St. Venant and Whitney approaches. Their idea is to approximate the dipolar source term of the EEG forward problem by a monopole distribution in the vicinity of the source location, see [20, 38, 54]. The subtraction source model adopts a different approach and deals with the singular source term indirectly. It relies on the assumption that there exists a small area around the source location where the conductivity is constant, denoted by *σ*_∞_. The potential and the conductivity can then be split into two contributions, a singularity contribution and a correction part: *u* = *u*_∞_ + *u*_*corr*_, and *σ* = *σ*_∞_ + *σ*_*corr*_. While *u*_∞_ can be computed analytically, the insertion of the above decomposition into the EEG forward problem results in a Poisson equation for the correction potential. This equation can be solved numerically to compute *u*_*corr*_ which can then be added to *u*_∞_ for the complete potential, see [3, 52].

The discretization of the EEG forward problem using FEM leads to a system *Ax* = *b* to be solved for $x\in R^{n}$, where $A\in R^{n\times n}$ denotes the *stiffness matrix*. The right-hand side vector $b\in R^{n}$ represents the source term and depends on the respective source modeling approach. In order to speed up the computation of the solution to the EEG forward problem, we first note that for the computation of a lead field matrix, it is not necessary to know the potential in the interior of the domain Ω, but only the evaluation at the sensor positions on the boundary. For a given source, this leads to a potential vector $\tilde{x}\in R^{p}$, where p∈N denotes the number of sensors. Given a solution $x\in R^{n}$, the evaluation can be represented by a linear map $R\in R^{p\times n}$ as $\tilde{x}=Rx$. Inserting *x* = *A*^−1^*b* and defining *T* ≔ *RA*^−1^ results in $Tb=\tilde{x}$. This means, once *T* is known, the EEG forward problem can be solved by computing the right-hand side vector *b* and performing a matrix-vector multiplication. The matrix $T\in R^{p\times n}$ is called the (EEG) *transfer matrix*. By exploiting the symmetry of the discrete operator *A*, the transfer matrix can be computed row-wise by solving *AT*^*t*^ = *R*^*t*^ using the *p* rows of *R* as the right-hand sides. Thus, for the computation of the transfer matrix, the linear system has to be solved once for every sensor location [12, 14, 57].

### MEG

The solution of the MEG forward problem directly follows from the solution of the respective EEG forward problem via the law of Biot-Savart [1, 58]. For a current distribution *j*, the magnetic field B at a position $y\in R^{3}$ is computed via

$$\begin{array}{c} B\left(y\right)=\frac{\mu_{0}}{4\pi }\int_{\Omega }j\left(x\right)\times \frac{y-x}{{\left\|y-x\right\|}^{3}}\text{d}x, \end{array}$$ (2)

where × denotes the three-dimensional cross product and *μ*_0_ the permeability of free space.

By splitting the current distribution *j* into the primary current *j*^*p*^ and secondary current *j*^*s*^ = −*σ*∇*u*, the magnetic field B in Eq (2) can be divided into the primary magnetic field *B*^*p*^ and the secondary magnetic field *B*^*s*^. While there is an analytical expression for *B*^*p*^ [1, 58], in order to compute *B*^*s*^ the integral expression

$$\begin{array}{c} \int_{\Omega }\sigma \left(x\right)\nabla u\left(x\right)\times \frac{y-x}{{\left\|y-x\right\|}^{3}}\text{d}x \end{array}$$

needs to be computed numerically. For the standard continuous Galerkin method (CG-FEM) this integral can be directly evaluated using the discrete representation of the potential *u*_*h*_. Results for the MEG approach for the discontinuous Galerkin method (DG-FEM) in [16] indicate that a direct usage of *σ*∇*u*_*h*_ leads to suboptimal accuracies. Instead, the numerical flux of the discontinuous Galerkin method should be used, see [16, 19]. Similarly to the EEG case, a transfer matrix can be derived which allows computing *B*^*s*^ at the sensors with a matrix-vector multiplication, instead of solving the EEG forward problem and computing the integral subsequently, see [19, 57]. Note that when using the subtraction approach, the resulting solution does not include the contributions of the singularity potential [16].

## Software structure and design

DUNEuro relies on the DUNE software toolbox which offers functionality such as grid implementations, function space discretizations and solvers. In this section, the dependency of the DUNEuro toolbox on the DUNE modules is explained in more detail. In addition, core concepts of the internal structure of our software are presented. These include the so-called driver interface which is the core user interface for the MATLAB and Python bindings. Additionally, the implementations of different solvers and source models are explained. Besides presenting the software design of these subcomponents, examples are given to highlight the extendibility of our software by giving examples on how to implement new features. Subsequently, we will describe an algorithm for localizing elements within a tessellation based on a global coordinate.

### DUNEuro and the DUNE framework

DUNE is an open-source C++ toolbox providing various functionality related to the numeric solution of partial differential equations [59]. It comprises different modules that offer well-defined distinct features as a separate entity but may depend on each other. Each separate module can be downloaded and installed, the program *dunecontrol* manages this process and the module dependencies. A list of DUNE modules that DUNEuro depends on as well as a short description of their functionality is provided in Table 2.

**Table 2:** Overview of DUNE and DUNEuro modules.

Module	Description
dune-common	common infrastructure
dune-geometry	grid elements and quadrature
dune-istl	sparse linear algebra
dune-localfunctions	local finite element spaces
dune-grid	grid interface
dune-uggrid	unstructured grids
dune-typetree	tree data structures
dune-functions	functions and function space bases
dune-pdelab	finite element assemblers and solvers
dune-udg (opt.)	unfitted discretizations
dune-tpmc (opt.)	marching cubes/simplex algorithm
duneuro	discretization of bioelectromagnetic equations
duneuro-matlab (opt.)	MATLAB interface
duneuro-py (opt.)	Python interface

The description of the DUNE modules is based on [59], for further details, see also [32–34, 48].

DUNEuro relies on several DUNE modules. The main functionality is provided by the core modules dune-common, dune-geometry, dune-istl, dune-localfunctions and dune-grid. For representing geometry-conforming tetrahedral and hexahedral meshes, we use the grid implementations provided by the dune-uggrid module [34]. The discretization of the partial differential equation makes use of the dune-pdelab module [35] that offers different discretization schemes along with appropriate finite elements allowing a rapid prototyping of new models. This module offers abstractions for the concept of a function space on a grid or for the linear operator used in the discretization. The implementation of the unfitted discontinuous Galerkin method is provided in the dune-udg module [60]. One component of the unfitted finite element methods is the integration over implicitly defined domains, which is performed using the C++ library tpmc (http://github.com/tpmc). Both libraries, dune-udg and dune-tpmc, are optional in case unfitted methods are applied. For the MATLAB or Python bindings of DUNEuro, the respective modules duneuro-matlab and duneuro-py can be installed in addition to the duneuro module itself. The compiled libraries can then be used to integrate the functionality of DUNEuro in any Python or MATLAB pipeline. Detailed step-by-step instructions on how to download and install these modules are provided in S1 Appendix.

### The EEG-MEG driver interface

As described above, there are several different discretization schemes available for solving the EEG forward problem and each scheme provides different source models. The finite element methods presented here can be split into two different categories: the *fitted* and *unfitted* discretization methods. The fitted category refers to a discretization method that uses a grid whose geometry is fitted to the model geometry. The basis of this approach is a VolumeConductor class that stores the grid along with the conductivity tensor of each grid element. Currently, there are two different fitted discretization schemes implemented in DUNEuro: the conforming Galerkin (CG-FEM) and the discontinuous Galerkin (DG-FEM) finite element methods [15, 16, 61]. Methods that also fall into this category but are not yet available are mixed finite element methods [46] or finite volume schemes. The unfitted category refers to a discretization method that uses a grid which is independent of the model geometry and employs the model geometry weakly. The model geometry is provided implicitly via level-set functions and considered in the weak formulation. Currently, the unfitted discontinuous Galerkin (UDG) method is implemented as a discretization scheme in the unfitted category [17].

**Fig 1:**
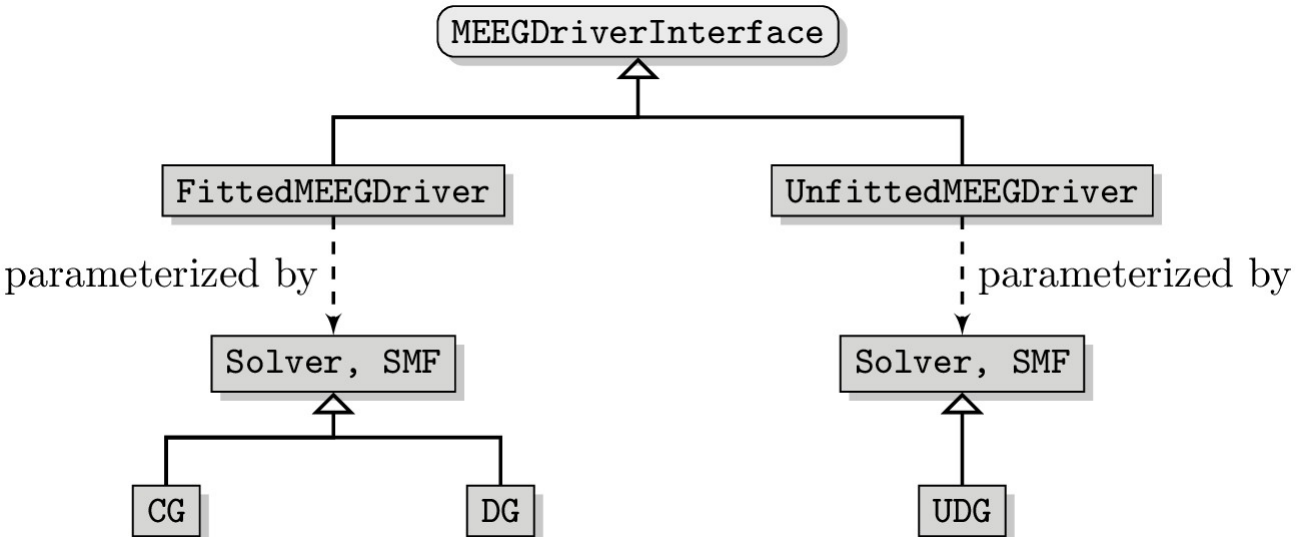
DUNEuro driver interface diagram. Diagram showing the structure of the driver interface and its implementations. The SourceModelFactory is abbreviated as SMF.

From the user perspective of a software framework, it should be simple and intuitive to change from a fitted to an unfitted discretization or between different discretization schemes within each category. For example, switching from CG-FEM to DG-FEM should not require fundamental changes in the user code. A further consideration when designing the interface of the software is the way the user will interact with it. As described in more detail below, we want to provide bindings to programming languages a potential user is already familiar with, such as Python or MATLAB. In order to simplify both, the overall user interface and the process of creating such bindings, we define a single coarse grained interface class to interact with the internal toolbox. This interface class is called the MEEGDriverInterface. It describes the general concepts of solving EEG and MEG forward problems. Each of the two discretization categories is implemented by its own driver class, the FittedMEEGDriver and the UnfittedMEEGDriver, respectively. Fig 1 shows a general diagram of the MEEGDriverInterface. For each category, the implementation of the discretization scheme is provided via two template parameters: a Solver and a SourceModelFactory. The purpose of the solver class is to bundle the handling of the stiffness matrix and the solution of the resulting linear system. The source model factory will construct source models whose purpose is the assembly of the right-hand side. Both the solver and the source model factory are further described below. The user of the toolbox will not directly interact with the implementation of the drivers, but only with the driver interface class.

### The solver and the source model factory

The purpose of the solver class is the assembly of the stiffness matrix and the solution of the linear system. It contains the discretization scheme as well as the necessary function spaces for representing discrete functions. The main interface method is a solve method which, given a right-hand side vector, solves Poisson’s equation and returns the discrete solution. Several forward problems in bioelectromagnetism, e.g., the EEG forward problem, electric [23, 62] or magnetic stimulations [63, 64] or the computation of a transfer matrix, mainly differ with respect to the right-hand side of the linear system. The solver class can thus be reused for any such purpose. By using a single solver class, the stiffness matrix has to be assembled only once and can be reused for further purposes. As the different discretization schemes differ in the way the matrix is stored, e.g., with respect to the blocking scheme of the matrix entries, this information is hidden from the interface. The purpose of the source model factory is to construct the different source models dynamically based on a configuration provided by the user. All source models provide a common interface which is described below.

We will illustrate the extendibility with respect to the discretization scheme using the example of a mixed finite element method [46]. Mixed-FEM is based on a first-order representation of Poisson’s equation and employs unknowns for both the potential and the electric current. It derives a weak formulation and uses scalar and vector-valued finite elements on a geometry-conforming grid as a discretization. It thus falls into the category of fitted discretization schemes. In order to use the described FittedMEEGDriver, one needs to provide two components: a MixedFEMSolver and a MixedFEMSourceModelFactory. The MixedFEMSolver contains the discretization of the stiffness matrix as well as the definition to solve the resulting linear system. The implementation of such a solver class is heavily based on the dune-pdelab module which contains, for example, the implementations of the local basis functions. The MixedFEMSourceModelFactory offers a method to create different source models for the Mixed-FEM approach, whose purpose is then to assemble the right-hand side vector for a given source position. In [46], two different source models have been presented: a *direct approach* and a *projected approach*. Finally, one has to provide means to evaluate a discrete solution at electrode positions along with the resulting right-hand side of the transfer matrix approach. Once the above-mentioned software components related to the solver and source models for the Mixed-FEM approach are implemented, the features of the driver, e.g., computing a transfer matrix or solving the EEG forward problem, are available.

**Fig 2:**
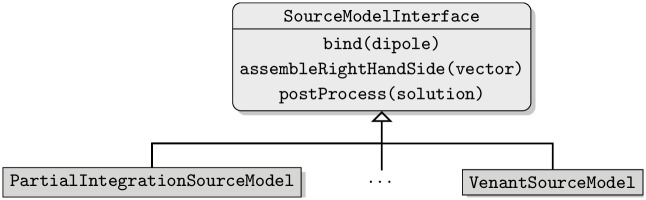
DUNEuro source model interface diagram. The structure of the source model interface and its implementations.

The common task of source models can be stated as: given a dipole position and a dipole moment, assemble the right-hand side vector. This right-hand side vector will then be passed on to the respective solver class described above. As there is still research ongoing and new source models are being developed, it should be easy to provide an additional source model without substantial modifications of the existing code. In addition, it should be possible to choose the source model at runtime, both for investigating the effects of different source models as well as ruling out the source model as a source of errors. Some source models, such as the subtraction approach, do not provide a right-hand side for the full potential, but need to apply an additional post-processing step to the resulting solution in order to obtain the full potential. For the subtraction approach, this post-processing step consists of adding the singularity potential *u*_∞_ to the correction potential *u*_*corr*_. As this post-processing step depends on the type of the source model and the user should have the option to turn off the post-processing, it is provided as a method of the source model interface. Fig 2 shows a diagram of the general SourceModelInterface along with its implementations.

When applying the transfer matrix, i.e., multiplying the transfer matrix *T* with the right-hand side vector *b*, the computational complexity depends on the number of non-zero entries in *b*. A main advantage of the direct source models such as the partial integration approach or the St. Venant approach is the sparsity of the right-hand side with only a few non-zero entries in *b*, typically independent of the mesh resolution. Indirect source models such as the subtraction approach, on the other hand, lead to a dense right-hand side vector. As a result, two different data types, i.e., sparse and dense vector containers are used to store the right-hand side vectors in order to reduce computation time for the direct approaches. In order to be able to handle both types for the right-hand side, the vector type is provided as a template parameter of the source model interface.

We will illustrate the extendibility with respect to the source models on the example of a modified subtraction approach for CG-FEM. In [18], a modification of the subtraction approach has been presented: the *localized subtraction approach*. It restricts the contribution of the singularity part of the potential to a patch around the source location. As the functions within a DG-FEM discretization can be discontinuous, they can directly capture the jump occurring at the boundary of the patch. For a CG-FEM discretization, such jumps can not be directly resolved and thus the localization scheme has to be modified. Instead of using a restriction of the singularity contribution to the patch, one can multiply the singularity contribution with a function that linearly interpolates within an interface zone of the patch between the singularity potential and zero. A source model implementing this localized subtraction approach would provide a class fulfilling the source model interface. Within the bind method in Fig 2, the local patch would be created and the linear interpolation in the interface zone could be constructed. The implementation of the assembleRightHandSide method contains the integration of the different model terms, resulting in the right-hand side. The postProcess method adds the singularity potential to the correction potential on the local patch.

### Element localization

A common subtask when assembling the right-hand side for a given dipole is the localization of the mesh element containing the dipole. For a sparse source model, this is especially relevant, as the time of assembling the right-hand side is usually constant, once the dipole element has been found. The complexity of the right-hand side assembly thus strongly depends on the complexity of the method that is used for finding the dipole element. The most straightforward approach is given by a linear search among the mesh elements. Assuming an ordering of the mesh elements, we evaluate for each element of the mesh if it contains the dipole position. Once the result of the evaluation is positive, we return this element. This algorithm has an average and worst-case complexity of $O(n_{m})$, where *n*_*m*_ denotes the number of the mesh elements.

A first step to speed up the localization can be found by using geometric information when iterating the mesh elements instead of using a fixed ordering [65]. The method presented here is called *edge-hopping*:

1. Start at a given mesh element and iterate over all faces of the current element.
2. Compute the relative position of the dipole location and the hyperplane induced by the face center and its outer normal.
3. If the dipole lies in normal direction, continue the search at step 1 with the neighboring element if such an element exists.
4. If the face has no neighboring element, the dipole lies outside of the mesh or the mesh is not convex. Terminate the search.
5. If the dipole lies in the opposite direction, continue at step 2 with the evaluation of the next face.
6. If the dipole lies on the inside of all faces of the current element, the dipole element has been found.

A requirement of the edge-hopping method is the convexity of the mesh, that is usually only fulfilled by the multi-layer sphere models, and not by the realistically shaped head models. However, as the algorithm monotonously moves closer to the dipole element, we only need convexity of the mesh in a sphere around the dipole location and the starting point of the iteration. As the considered sources lie in the gray matter compartment, that is completely enclosed by the skin, we can easily find such a sphere around the source locations if the starting location is close to the source position. In order to find an element that is close to the source location, we insert the element centers into a *k-d Tree*, that is a data structure to efficiently perform nearest-neighbor searches [66]. It does so by recursively splitting the set of element centers along the Cartesian directions. Even though the center of the element which is closest to the dipole location does not have to belong to the element containing the dipole, it can be assumed to be close to the desired element. It thus offers an efficient starting point for the edge-hopping algorithm.

## Interface to scripting languages

In this section we describe the interaction of a user with the DUNEuro library. In general, a common approach is to provide a compiled binary executable that the user is able to call directly. This executable would then load the data provided by the user from the hard disk, perform the desired computation and write the computed result back to the hard disk. As different users might want to perform different sets of computations, the computations to be performed can be configured by the user, either through command line parameters or through a configuration file. An advantage of this approach is its very simple and straightforward usage, similar to any other executable on the operating system. There is no need for additional packages or additional software and the executable can be used directly by the user. However, the computation of the solution to the forward problems is usually only a small part in a longer pipeline for source analysis. This pipeline usually consists of the data measurements and pre-processing steps and the forward solution is part of an inverse estimation process. When using the library directly in an executable, one has to provide methods for reading any input data as well as writing out the resulting output. Similarly, the configuration has to be transferred to the executable by the user. As a consequence, the functionality of DUNEuro is currently not provided as an executable.

Instead, a different approach was adopted by offering bindings to scripting languages, i.e., MATLAB (The Math Works Inc., Natick, Massachusetts, United States; https://www.mathworks.com) and Python (Python Software Foundation, https://www.python.org). For both languages there are already existing software frameworks for processing EEG and MEG data [24–27]. Thus, by providing direct bindings one can include the forward modeling approach directly into an existing analysis pipeline. An example for a similar integration is presented in [30], where the authors introduce a pipeline for performing EEG source analysis using the conforming finite element method together with the classical St. Venant source model. The forward models are implemented using the SimBio software (https://www.mrt.uni-jena.de/simbio) and integrated into the MATLAB-based FieldTrip-toolbox [24].

**Fig 3:**
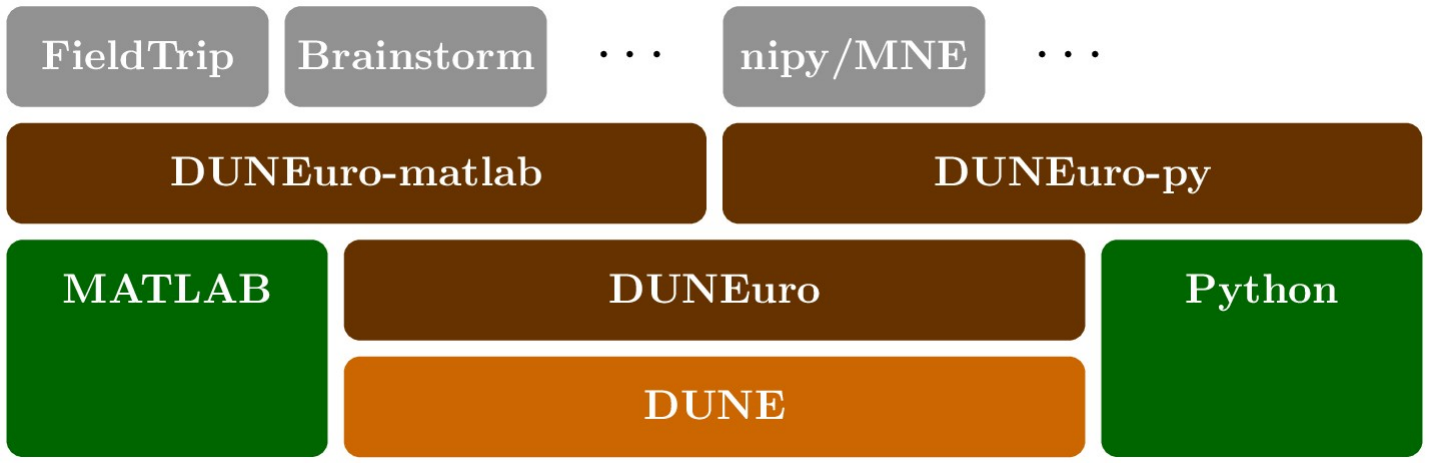
Modular structure. Relation of the DUNEuro modules with respect to DUNE, external software and downstream libraries.

DUNEuro builds upon several existing modules of the software toolbox DUNE and the core functionality is provided by the C++ module duneuro. Bindings to the MATLAB and Python scripting languages are provided in separate modules: duneuro-py and duneuro-matlab, respectively. A structural overview of DUNEuro and its interface modules with respect to DUNE, external software and downstream libraries is illustrated in Fig 3. The purpose of both modules is to translate the input data given as data structures in the respective programming language and translate them into the C++ counterparts. For some cases, this translation can be performed without copying any data, which is especially relevant for large matrices such as the transfer matrix. An example of the driver construction in a Python script is shown in Listing 1. The configuration of the discretization is provided as a Python dictionary. One possible option to define the head volume conductor model is via an input file for the mesh, e.g., using the gmsh file extension .msh (see https://gmsh.info) in combination with a plain text file containing the isotropic conductivity values. Alternatively, the mesh can also be provided directly by specifying the vertices, elements, tissue labels and conductivity tensors. An overview of different options and parameters to pass the volume conductor in case of fitted and unfitted FEM discretizations is provided in the GitLab Wiki. A detailed description of input and output parameters for the functionality provided by the driver interface is also provided. See also S2 Appendix for example data and scripts. Note that the discretization method, in this case cg, is provided as a parameter in the configuration. By changing it to dg and adding the necessary additional parameters such as the penalty parameter *η*, one can directly use the discontinuous Galerkin method through the same interface. Thus, once a user is able to use the DUNEuro library for any discretization method, a switch to a different discretization method can be directly performed. Listing 2 shows the same construction of the driver object as in Listing 1 using the MATLAB interface. The general structure of the MATLAB script is similar to the Python script. The main differences are the use of MATLAB syntax and the replacement of the Python dictionary by a MATLAB structure array. Even though the wrapper code for creating the driver object is different, both scripting languages interface the C++ library and use the same code base.

**Listing 1**. Example Python script for creating an MEEGDriver.

import duneuropy as dp

config = {

‘type’: ‘fitted’,

‘solver_type’: ‘cg’,

‘element_type’: ‘tetrahedron’,

‘volume_conductor’: {

‘grid.filename’: ‘path/to/grid.msh’,

‘tensors.filename’: ‘path/to/tensors.dat’

}

}

driver = dp.MEEGDriver3d(config)

**Listing 2**. Example MATLAB script for creating an MEEGDriver.

cfg = [];

cfg.type = ‘fitted’;

cfg.solver_type = ‘cg’;

cfg.element_type = ‘tetrahedron’;

cfg.volume_conductor.grid.filename = ‘path/to/grid.msh’;

cfg.volume_conductor.tensors.filename = ‘path/to/tensors.dat’;

driver = duneuro_meeg(cfg);

## Example: Source analysis of somatosensory evoked potentials

**Fig 4:**
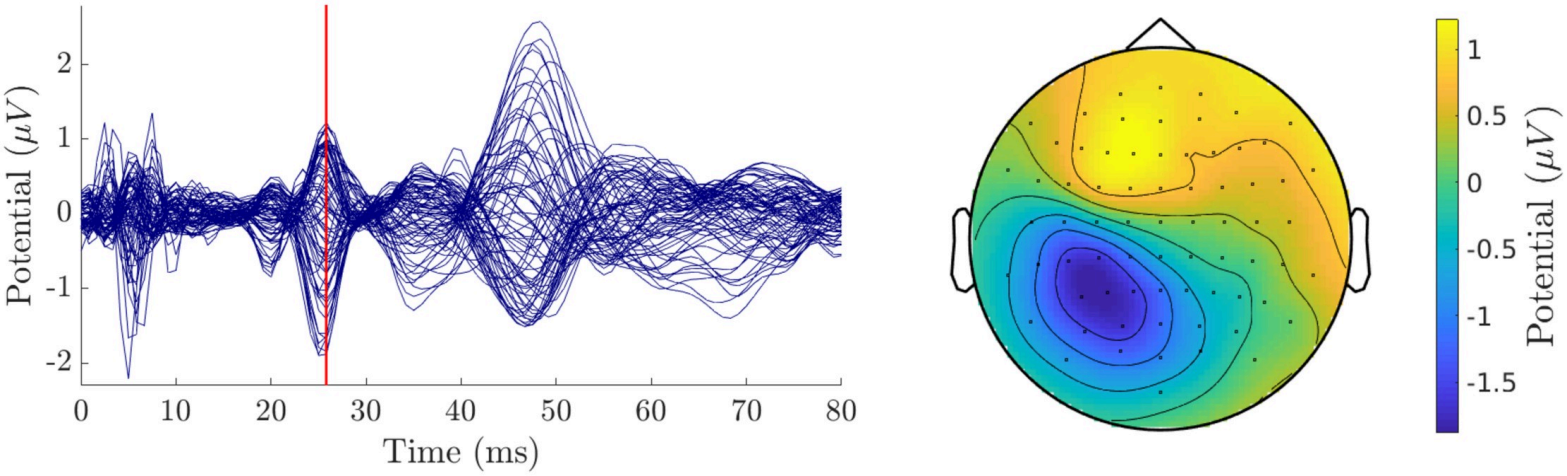
Practical application: Preprocessing. Left: Butterfly plot of the somatosensory evoked potentials. The vertical red line indicates the 25.8 ms time point. Right: topography plot of the averaged potential at the electrodes for *t* = 25.8 ms.

As a practical example how forward solutions (in this case UDG-FEM) computed with DUNEuro can be embedded in an entire source localization pipeline, we reconstruct the P20/N20 peak of somatosensory evoked potentials in the primary somatosensory cortex. A right-handed, 49 years old, male volunteer participated in a somatosensory evoked potential (SEP) EEG recording of an electric stimulation of the right median nerve, available from [67]. The participant had no history of psychiatric or neurological disorders and had given written informed consent before the experiment. All procedures had been approved by the ethics committee of the University of Erlangen, Faculty of Medicine on 10.05.2011 (Ref. No. 4453). The EEG was measured using 74 electrodes, whose positions where digitized using a Polhemus device (FASTRAK, Polhemus Incorporated, Colchester, Vermont, USA). The medianus stimulation was done in supine position in order to reduce modeling errors due to brain movement, because the corresponding MRI for head volume conductor modeling was also measured in supine position [68]. In total, 1200 stimuli were applied, the inter-stimulus interval was randomized in the range of 350 ms to 450 ms. The EEG data was preprocessed using FieldTrip [24] with a band-pass filter from 20 Hz to 250 Hz and notch-filters at 50 Hz and harmonics to reduce power-line noise. After removing one bad channel (P7) the remaining trials where averaged to produce the evoked potential data. Fig 4 shows a butterfly plot of the resulting time series of the averaged potentials as well as a topography plot of the potential measured at the electrodes at the peak of the P20 component at the time point of 25.8 ms due to the time delay until the stimulus arrives at the median nerve. The first milliseconds show the stimulation artifact. At about 5.8 ms, the action potential is on the median nerve and starts traveling up to produce 20 ms later at 25.8 ms the P20 component.

**Fig 5:**
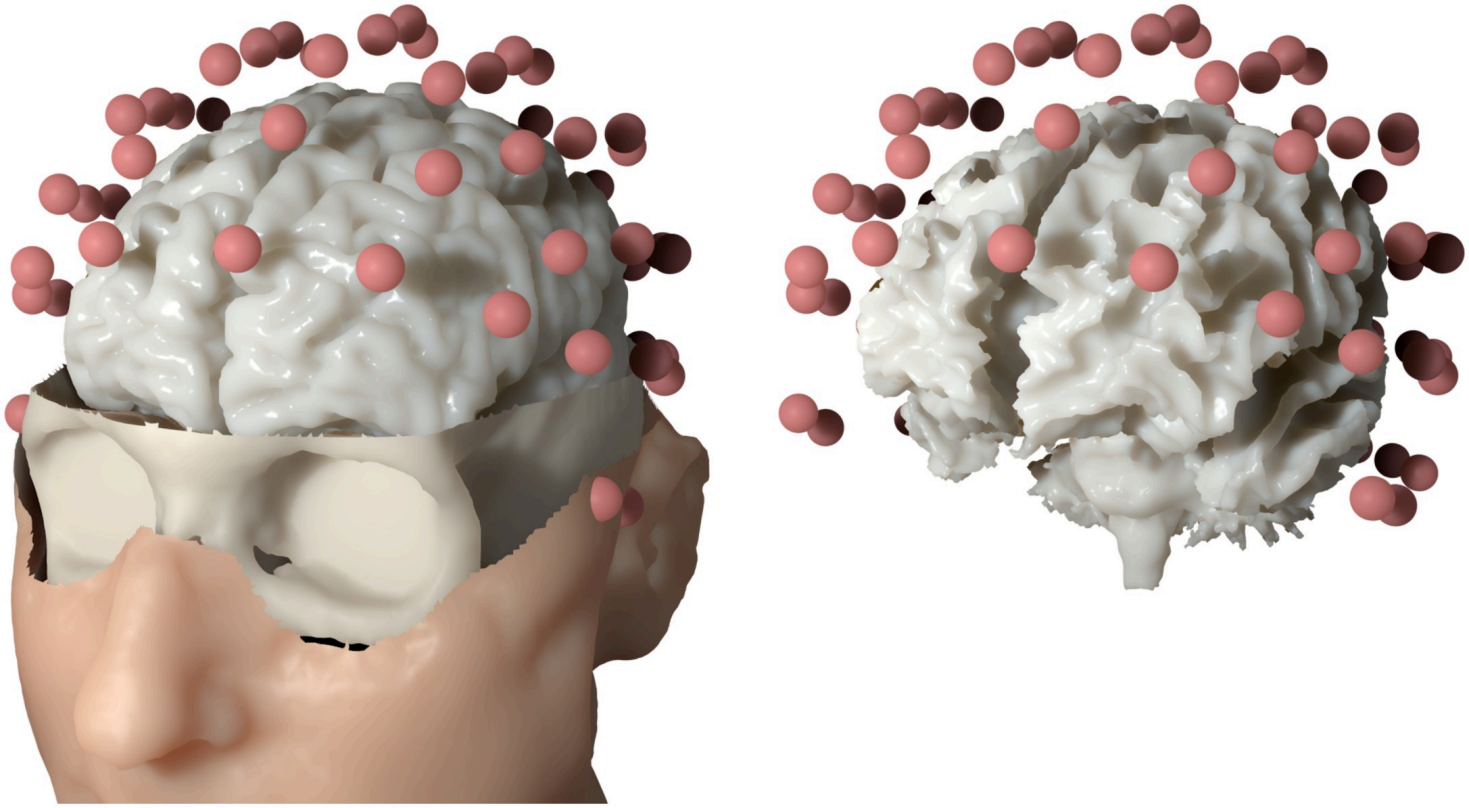
Practical application: Realistic head model. Left: skin, skull and gray matter surfaces of the six-compartment isotropic head model along with the electrode montage used in the practical example. Right: source space relative to the electrode positions.

Using a 3 T MRI scanner (Siemens Medical Solutions, Erlangen, Germany), T1-weighted and T2-weighted MRI sequences were measured. Based on these MR images, a six-compartment voxel segmentation has been constructed, distinguishing between skin, skull compacta, skull spongiosa, cerebrospinal fluid, gray matter and white matter using SPM12 (http://www.fil.ion.ucl.ac.uk/spm/software/spm12) via FieldTrip [24], FSL [69] and internal MATLAB routines. We extracted surfaces from this voxel segmentation to distinguish between the different tissue compartments. To smooth the surfaces while maintaining the available information from the voxel segmentation, we applied an anti aliasing algorithm created for binary voxel images presented in [70]. The resulting smoothed surfaces are represented as level-set functions and are available from [67]. The digitized electrodes were registered to the head surface using landmark-based rigid parametric registration. Especially in occipital and inferior regions, due to the lying position of the subject during MRI measurement, the gray matter compartment touches the inner skull surface, which also motivates the use of unfitted FEM [71]. From Fig 4 we see a clear dipolar pattern in the topography plot. To estimate the location of the dipole, we performed a single dipole deviation scan using a normal constraint for dipole orientation on the source space, i.e., a set of source locations within the gray matter compartment [58]. The source space was created using a weighted sum of level-set functions for gray and white matter as *α*Φ_wm_ + (1 − *α*)Φ_gm_ with *α* = 0.8. Like the levels-sets for white and gray matter, the resulting level-set for the source space is then given as a three-dimensional array of signed-distance values. This level-set function was discretized using the marching cubes algorithm presented in [72], which resulted in 256 134 source locations. For each location, we computed the dipole orientation normal to the surface of the source space. Fig 5 shows the skin, skull and gray matter surfaces and the electrode positions as well as the source space that was used in the example computation.

Using the level-set functions, we constructed an unfitted FEM head model and computed the EEG transfer matrix for all electrode positions using the presented DUNEuro toolbox. With this transfer matrix, we computed the EEG forward solution for all dipole positions with the fixed orientation and unit strength using the partial integration source model [14, 56]. The optimal strength *s* with respect to a given measurement $m\in R^{p}$ for a dipole with the leadfield $l\in R^{p}$, where p∈N denotes the number of sensors, can be obtained by minimizing ‖*ls* − *m*‖_2_ over *s*. The resulting optimal strength for reproducing the measured data can be computed by multiplying the pseudo-inverse of *l* with the measurement vector *m*. As *l* consists of a single column due to the fixed dipole orientation, this reduces to

$$\begin{array}{c} s=\max(\frac{{\left\langle l,m\right\rangle }_{2}}{{\left\|l\right\|}_{2}^{2}},0). \end{array}$$

The maximum with 0 is used in order to restrict the solution along the respective positive normal direction because the P20/N20 component is assumed to be located in Brodmann area 3b of the somatosensory SI cortex pointing out of the cortex to produce a frontal positivity [73, 74]. This strength is embedded into the goodness of fit (GOF) measure that is defined as

$$\begin{array}{c} \mathrm{GOF}=1-\frac{{\left\|ls-m\right\|}_{2}^{2}}{{\left\|m\right\|}_{2}^{2}} \end{array}$$

and measures the ability of the numerical solution to reproduce the measured data. If the data can be exactly reproduced, the GOF has a value of 1. In the case of a single dipole deviation scan this includes how well the data can be represented as a single dipole. Former results have shown that the single dipole source model is appropriate for the reconstruction of the early somatosensory response [73–76].

**Fig 6:**
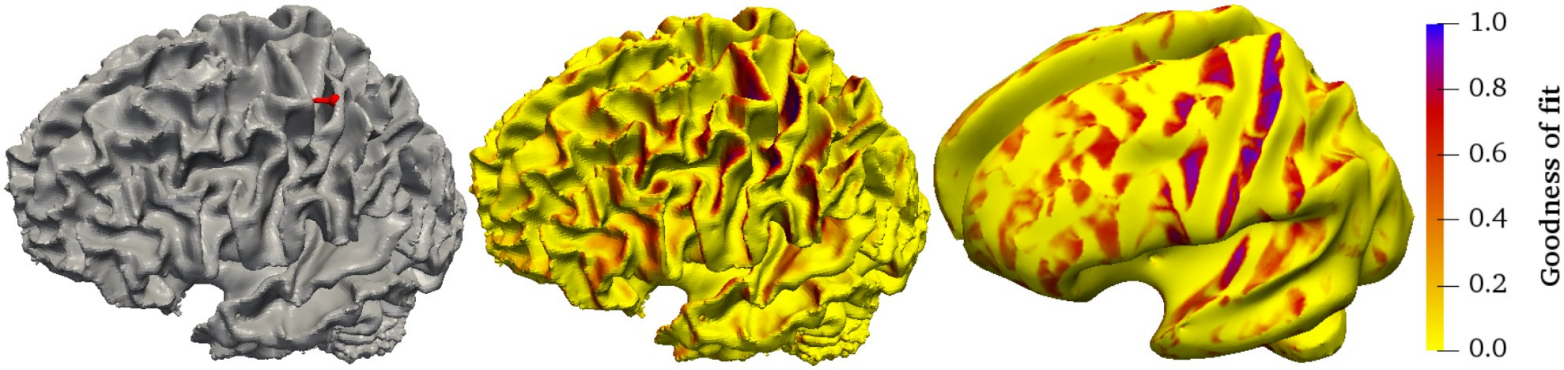
Practical application: Source reconstruction. Left: Reconstructed source (red) of the P20 component and its location within the source space. Middle: Distribution of the GOF measure on the source space. A darker color indicates a higher GOF. Right: GOF measure on an inflated model of the source space.

The source of the P20/N20 response was reconstructed in the primary somatosensory cortex in the wall of the post-central gyrus with a GOF of 0.962 and with a mainly tangential orientation, which reproduces findings of [73–76]. Fig 6 shows the source embedded in the source space and the distribution of the GOF measure on a realistic and an inflated model of the source space. We see that the GOF measure is higher for source locations on the gyral walls with a tangentially oriented normal vector and that the higher values are located close to the central sulcus. Overall, the GOF measure shows a smooth distribution in these areas while being sensitive to orientation changes.

## Summary and outlook

In this paper we presented the DUNEuro software, a toolbox for solving forward problems in bioelectromagnetism. We provided a general description of the toolbox as well as detailed information about the main concepts. Short examples showed the extendibility of the different subcomponents. We presented a method to efficiently localize positions within a given mesh and described bindings of the library to scripting languages. Finally, the practical usability of the library was demonstrated by a source analysis of experimental data of a somatosensory stimulation.

The DUNEuro toolbox offers a flexible and efficient way to solve the EEG/MEG forward problems numerically using modern FEM approaches. There are several open goals regarding the software implementation. Foremost, a direct comparison with existing tools for computing forward solutions for EEG and MEG, such as the SimBio toolbox, is currently performed [77]. Similar to the latter toolbox, a closer integration into existing EEG/MEG source analysis frameworks [24–27] would further facilitate its usability. An integration into the Brainstorm toolbox [25], for instance, is currently under development (https://neuroimage.usc.edu/brainstorm/Tutorials/Duneuro) [78]. This would then also allow to evaluate the advantages and disadvantages of the new FEM methods that are now available through the DUNEuro code in practical applications. Additionally, this integration would offer the use of DUNEuro for different inverse approaches. This would also allow the reconstruction of a complete localized time course of the somatosensory responses using forward solutions of DUNEuro as well as comparisons of these source analysis results to other methods (e.g., BEM). Several other forward problems, e.g., electric or magnetic brain stimulations whose mathematical formulations are closely related to the EEG/MEG forward problems due to Helmholtz reciprocity [21–23] are already partly implemented, but their support should be improved and evaluated. Of special interest would then be a connection to optimization procedures for transcranial direct current stimulation [23, 79, 80]. Even though DUNEuro already relies on the testing infrastructure of the DUNE framework, further work on a complete testing framework using continuous integration is necessary in order to improve the stability of the code base and ensure the reliability of the results even under future modifications. As DUNEuro is further developed and used, the documentation of its features and user interfaces is steadily extended.

## Supporting information

S1 Appendix Installation instructions. A detailed description is provided how to install the DUNEuro software toolbox. The DUNE and DUNEuro modules are downloaded using an auxiliary bash script and option files are provided which are used in the compilation process.
(ZIP)

S2 Appendix Example data and scripts. Example scripts in Python and MATLAB are provided to give an overview of the numerous features DUNEuro offers. For the volume conductor head model, different spherical four-compartment models and two different realistic six-compartment head models are used. A detailed description is given that lists all provided data files and scripts and explains the different steps for an EEG forward calculation using DUNEuro. Different FEM discretizations, source models, and solution approaches (direct or via the transfer matrix approach) are used and explained in detail.
(ZIP)
