# Supplementary material for: DUNEuro—A software toolbox for forward modeling in bioelectromagnetism
Source: PLoS One. 2021 Jun 4;16(6):e0252431. doi: 10.1371/journal.pone.0252431 (PMC8177522; doi:10.1371/journal.pone.0252431)
Supplement: S1 Appendix — A detailed description is provided how to install the DUNEuro software toolbox. The DUNE and DUNEuro modules are downloaded using an auxiliary bash script and option files are provided which are used in the compilation process. (ZIP) [file pone.0252431.s001.zip › S1_installation_instructions/installation_instructions.pdf]

Installation instructions

In the following, we will describe how to download and compile the DUNEuro software toolbox along with its Python and Matlab bindings on a Linux computer. The instructions assume root access on a Ubuntu system and have been tested for Ubuntu 18.04 and 20.04 using g++ compiler versions 7.5.0 and 9.3.0, respectively. They might require minor adjustments related to the installation of external packages if a different distribution is used. We will use the 2.6-release versions of DUNE and DUNEuro, please have a look at the DUNEuro homepage `http://www.duneuro.org` to see if there are installation instructions using newer versions.

1 Prerequisites

In a first step, we need to make sure that all necessary tools and libraries are installed. To do so, the following command can be run in a terminal (Ctrl + Alt +T):

```
sudo apt install build-essential cmake git libeigen3-dev \
  libpython3-dev libsuitesparse-dev
```

This command installs several required packages, such as `build-essential`. For Ubuntu 20.04 we additionally require the package `gfortran`, which can be installed in the same way.

```
sudo apt install gfortran
```

There are several other useful optional packages which can be installed. In order to take advantage of multi-threading for the computation and multiplication with the transfer matrix, the Intel® Threading Building Blocks (TBB) library (`github.com/intel/tbb`) can be additionally installed via the package `libtbb-dev`. For the direct solution of large systems of linear equations we also recommend to install the `libsuperlu-dev` package. Furthermore,

DUNEuro can generate output which can be visualized using the ParaView software (`https://www.paraview.org/`). In order to install these additional packages, the following command can be used:

```
sudo apt install libtbb-dev libsuperlu-dev paraview
```

Optionally, if the unfitted methods in DUNEuro are required, we need to install the tpmc (`https://github.com/tpmc/tpmc`) library. The easiest way to install tpmc is via pip:

```
sudo apt install python3-pip python3-numpy
sudo python3 -m pip install git+https://github.com/tpmc/tpmc.git
```

2 Download

The DUNE and DUNEuro modules are hosted in a GitLab repository (`https://gitlab.dune-project.org/`). We will use the provided helper script **clone.sh** which will assist in downloading the 2.6-release versions of DUNE and DUNEuro. In this script, some lines are optional, depending on the required methods. If the bindings to Matlab or Python via the modules `duneuro-matlab` or `duneuro-py` are not required, the correspondingly marked line in the script should be deleted. Similarly, the modules `dune-tpmc` and `dune-udg` are only required for the unfitted methods (UDG-FEM), if not required the marked line for downloading `dune-tpmc` can be deleted accordingly.

If unfitted methods are needed, download the `dune-udg` module from `https://www.dune-project.org/modules/dune-udg/` and extract it in the same folder that will be used for the other modules.

After moving the clone script to this folder and navigating to it in the terminal using the *cd* command, the clone script needs to be made executable, before it can be run:

```
chmod +x clone.sh
./clone.sh
```

This command will execute the clone script, which uses the *git clone* command to create local copies of the respective git repositories. Depending on the internet connection, the cloning might take a few seconds. Afterwards, the folders of all modules are located in the current directory.

3 Compilation

After the code has been downloaded, we can now compile it. All modules of DUNE and DUNEuro within the current directory will be compiled. Therefore, in order to avoid errors in the compilation process, e.g., during compilation of `duneuro-matlab` if Matlab cannot be found, unintentionally downloaded modules which are not supposed to be compiled should be deleted before continuing.

The attached file **config_release.opts** contains options and paths that are used for the compilation process and might need to be adapted. If DUNEuro-matlab is required, the path to the Matlab distribution should be indicated using the following flag:

```
-DMatlab_ROOT_DIR=/path/to/matlab \
```

While doing so, it is important that the trailing backslash '\' remains intact and is the last character in the line. If DUNEuro-matlab is not required, this line can be deleted. Similarly, if the unfitted methods are required, the path to the tpmc installation needs to be indicated (the parent folder can be identified using '`pip3 show tpmc`').

```
-DCMAKE_PREFIX_PATH=/path/to/tpmc \
```

If not applicable, this line can be deleted. If the computer has less than 16GB of RAM, the last line should be modified and '-j2' should be replaced by '-j1' which will reduce the number of cores that are used for parallel compilation.

In order to compile DUNEuro, we use the *dunecontrol* tool provided by the DUNE library. As parameters, we pass the options file that we modified above and the directory where all generated files should be stored, note the backquote characters (`) around *pwd* used for the build directory, which should not be confused with an apostrophe.

```
dune-common/bin/dunecontrol --opts=config_release.opts \
  --builddir=`pwd`/build-release all
```

More information about the *dunecontrol* command and its options can be found via:

```
dune-common/bin/dunecontrol --help
```

The execution of the command above will create a directory 'build-release' in the current directory which will contain the compiled library, once the compilation is finished. This compilation process will take some minutes, depending on the machine characteristics, the settings and the modules compiled.

After a successful compilation of `duneuro-py`, a file 'duneuropy.so' should be located in the 'build-release/duneuro-py/src' directory. Correspondingly, the file 'duneuro_matlab.mexa64' should be found in the 'build-release/duneuro-matlab/src' directory in case `duneuro-matlab` was compiled.

In case DUNEuro should be compiled for debugging, the steps described above need to be repeated for the **config_debug.opts** script by inserting the correct paths and running *dunecontrol* with this modified file as input.

```
dune-common/bin/dunecontrol --opts=config_debug.opts \
  --builddir=`pwd`/build-debug all
```

This will create a new folder 'build-debug' in the current directory.

4 Python and Matlab interfaces

In order to use the Python interface of DUNEuro, we need to tell Python where to find the compiled `duneuro-py` library prior to executing any Python script.

```
export PYTHONPATH=$PYTHONPATH:/path/to/build/duneuro-py/src
```

For the Matlab interface, when starting Matlab, depending on the system architecture and Matlab version, the path to the *blas* version as well as the path to the *libstdc++* library that were used to link DUNEuro may need to be indicated. This can be done using the following (adapted) command in the Matlab folder:

```
BLAS_VERSION=/usr/lib/libblas.so \
  LD_PRELOAD=/usr/lib/x86_64-linux-gnu/libstdc++.so.6 \
  ./matlab
```

As a next step, the **Example scripts** will provide first insights into the features DUNEuro offers.
