# Supplementary material for: DUNEuro—A software toolbox for forward modeling in bioelectromagnetism
Source: PLoS One. 2021 Jun 4;16(6):e0252431. doi: 10.1371/journal.pone.0252431 (PMC8177522; doi:10.1371/journal.pone.0252431)
Supplement: S2 Appendix — Example scripts in Python and MATLAB are provided to give an overview of the numerous features DUNEuro offers. For the volume conductor head model, different spherical four-compartment models and two different realistic six-compartment head models are used. A detailed description is given that lists all provided data files and scripts and explains the different steps for an EEG forward calculation using DUNEuro. Different FEM discretizations, source models, and solution approaches (direct or via the transfer matrix approach) are used and explained in detail. (ZIP) [file pone.0252431.s002.zip › S2_example_data_and_scripts/example_scripts.pdf]

Example scripts

In the following, we will illustrate how to use different features of DUNEuro from the Python and MATLAB interfaces by explaining several example scripts in detail. In these scripts, the numerical EEG forward solution is computed using different finite element method (FEM) discretizations, source models as well as different head models. We will also demonstrate how to write out the mesh and solution for visualization using the ParaView software [1] (`https://www.paraview.org/`). The examples are not exhaustive, but are designed to give an overview of different features that DUNEuro offers. The GitLab repository of DUNEuro contains a Wiki with more detailed documentation. This includes an overview of possible options and parameters for constructing the driver for fitted and unfitted methods, and an overview of functions the driver interface offers with their required input (e.g., source model parameters) and their output. In Section 1, we will describe different head models that are used in the example scripts. On the one hand, we use a spherical head model with four compartments. On the other hand, two different six compartment realistic head models are used to compute the EEG forward solution: a tetrahedral volumetric mesh and a head representation by level set functions. In Section 2, we describe the main steps when computing forward solutions using DUNEuro. Both for the spherical and realistic cases, we explain how the DUNEuro interface works for different FEM discretizations, source models and solution approaches (direct or transfer matrix approach). To run the example scripts, the DUNEuro toolbox needs to be installed following the procedure described in the **Installation instructions**. In order to use the Python bindings, the module `duneuro-py` needs to be successfully installed, so that the library ‘duneuropy.so‘ is located in the build folder. For the MATLAB bindings the file ‘duneuro_matlab.mexa64‘ is needed which will be in the build folder after successfully compiling the `duneuro-matlab` module. The choice of discretization method, the fineness of the mesh and the number of sensors determine the memory requirements for the computer used. For a standard Lagrangian

or Continuous Galerkin (CG-FEM) computation in a realistic head model we recommend at least 16 GB of RAM.

1 Example datasets

We will first give an overview of the different head models we use within the example scripts. These either fall in the category of spherical head models or realistic head models. For each model, additional input (e.g., electrode positions, test dipoles, ...) is provided.

1.1 Spherical head model

We use a 4-layer head model centered at [127 127 127] with four concentric spheres corresponding to the tissues of brain (78 mm radius, 0.33 S/m conductivity), cerebrospinal fluid (80 mm, 1.79 S/m), skull (86 mm, 0.01 S/m) and scalp (92 mm, 0.43 S/m). We use a tetrahedral and a hexahedral volumetric mesh as well as an unfitted representation of this geometry. Additionally, 70 electrode positions are used which are approximately uniformly distributed on the outer scalp surface. At each of 9 eccentricities ranging from 0.1 to 0.9 relative to the brain surface, 100 dipoles are randomly chosen with a tangential orientation. A visualization of the tetrahedral volumetric spherical mesh as well as an unfitted representation of the spherical head model is presented in Fig 1.

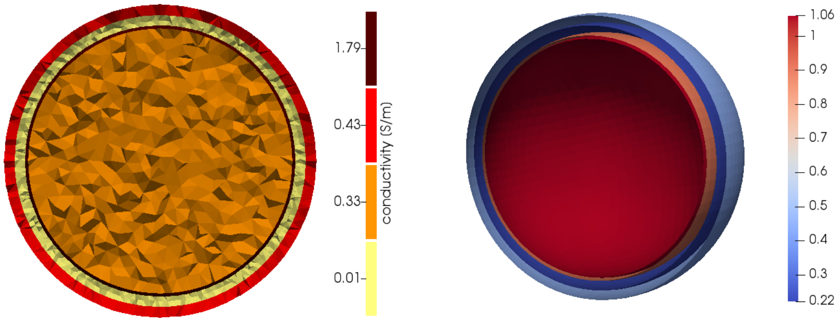

Figure 1. Spherical four-compartment head models: Tetrahedral volumetric mesh (left) and unfitted head model (right), here the conductivity on the internal tissue boundaries is shown as the mean between the conductivities of the two adjacent tissues.

The input files included in this appendix for the spherical head model are the following:

- **sphere_tet_mesh_4c.msh**: a tetrahedral mesh in gmsh [2] (`https://gmsh.info`) format, which contains 54 771 nodes and 306 439 elements as well as labels (0-3) for the physical entity indicating to which tissue an element belongs in the fourth column in the element list

- **sphere_4c.cond**: the conductivity values for each tissue type (corresponding to the label indices in the mesh files)
- **sphere_electrodes.txt**: 70 approximately uniformly distributed electrode positions on the outer surface
- **sphere_dipoles.txt**: 100 tangential dipoles at nine different eccentricities, the first three columns refer to the dipole position, the last three to the dipole moment
- **sphere_eeg_analytical.txt**: quasi-analytical EEG solution for our spherical four-compartment head model, where each row corresponds to the potential at an electrode for the 900 test dipoles, computed using the series expansion formulas following [3] via the software tool simbiosphere (`https://gitlab.dune-project.org/duneuro/simbiosphere`)

1.2 Realistic head model

In our example scripts, we use two different realistic six-compartment head models, which can be downloaded from [4]. Additionally required input data (e.g., electrode positions) are provided in this attachment. Both realistic models consist of the tissues scalp (0.43 S/m conductivity), skull compacta (0.0042 S/m), skull spongiosa (0.01512 S/m), cerebrospinal fluid (1.79 S/m), gray matter (0.33 S/m) and white matter (0.14 S/m). In the first model, the head is represented by a volumetric tetrahedral mesh, while the second model provides level sets for each tissue boundary. Both models are visualized in Fig 2. An overview of the input files related to the realistic head models is provided in the following.

1.2.1 Tetrahedral volumetric mesh

- **realistic_tet_mesh_6c.msh**: a tetrahedral mesh in gmsh [2] format, which contains 885 214 nodes and 5 335 615 elements as well as labels (0-5) for the physical entity indicating to which tissue an element belongs in the fourth column in the element list, provided at [4]
- **realistic_6c.cond**: conductivity values for each tissue type (corresponding to the label indices in the mesh file)
- **realistic_electrodes_fitted.txt**: 73 realistic electrode positions from an EEG measurement (EASYCAP GmbH, Herrsching, Germany), for which the positions were digitized using a Polhemus device (FASTRAK, Polhemus Incorporated, Colchester, Vermont, U.S.A.)

1.2.2 Unfitted head model

- **{skin, skull_compacta, skull_spongiosa, CSF, gray, white}.npy**: level sets representing the surfaces of each of the six tissue types: N^3-dimensional arrays (N=257) which discretize the background mesh and

contain signed distance values which are 0 at the surface, negative within and positive outside the surface, available at [4]

- **realistic_electrodes_unfitted.txt**: 73 realistic electrode positions from an EEG measurement (EASYCAP GmbH, Herrsching, Germany), for which the positions were digitized using a Polhemus device (FASTRAK, Polhemus Incorporated, Colchester, Vermont, U.S.A.)

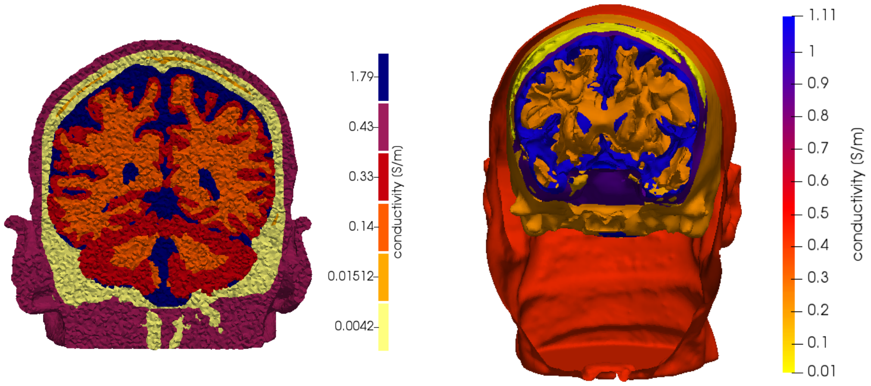

Figure 2. Coronal views of clipped realistic six-compartment head models: Tetrahedral volumetric mesh (left) and unfitted head model (right), here the conductivity on the internal tissue boundaries is shown as the mean between the conductivities of the two adjacent tissues.

2 Example scripts

We will now demonstrate how to use some of the features of DUNEuro using different example scripts. First, the general structure of a DUNEuro script which computes the EEG forward solution, is presented. Second, an overview of the provided example scripts is given, which either use the spherical or realistic head models. Third, we provide detailed explanations for a typical application: using CG-FEM to compute the EEG forward solution with different source models and the transfer matrix approach. In the following sections, some other features such as different FEM discretizations, will be addressed.

2.1 Main steps for computing EEG forward solutions with DUNEuro

Computing the EEG forward solutions with the DUNEuro toolbox generally consists of the following steps:

I) *DUNEuro Interface*: In order to be able to use the DUNEuro toolbox from Python/MATLAB, we need to import the duneuropy library (Python) or

add the path to our MEX file (MATLAB), which are contained in the build folder after a successful compilation of DUNEuro.

II) *Input*: The input files for our forward computation are defined (FE mesh, tissue conductivities, sources, electrodes,...). Note that the physical units of the input data provided define the output unit and should therefore be consistent, as DUNEuro currently does not check or convert units (e.g., if the dipole moments are provided in Amm, the geometry data in mm and the conductivities in S/mm, the resulting EEG potential has the unit V).

III) *Driver*: The driver is the common interface which is necessary to carry out the next steps. In order to create the driver, we need to specify the parameters related to our FEM discretization and the volume conductor head model.

IV) *Sensors*: The sensor characteristics are read and passed to DUNEuro.

V) *Forward solution*: The computation of the forward solution can be done using one of the following approaches

   (a) *Transfer matrix approach*: This case is advisable in case the dipoles outnumber the sensors, which is often the case in EEG/MEG source analysis. In this approach, the transfer matrix is computed in a first step, and applied afterwards in order to compute the forward solution at the sensor positions. In this second step, the source model needs to be specified and for each dipole, the right hand side is internally computed based on the source model configurations.

   (b) *Direct approach*: The forward solution is computed directly for each degree of freedom within the model, and evaluated at the sensor positions afterwards. This option is usually chosen for a small set of dipoles, e.g., for visualization purposes.

VI) *Postprocessing* (optional): Output for visualization with ParaView [1] can be created. Additionally, the computed leadfields can be further used for source analysis. In the spherical case, the numerical solutions can also be compared to the (quasi-)analytical solutions.

Due to this modular structure, single components can be easily modified, while the rest remains unchanged. For instance, if the user wants to employ a different source model, only the source model configuration in step V) needs to be adjusted, while the rest of the code remains unchanged.

2.2 Overview of example scripts

We will now demonstrate how to use some of the features of DUNEuro. Different fitted and unfitted FEM discretizations, source models, input meshes and solution approaches (direct or using the transfer matrix approach) will be exemplarily presented. Additionally, we will show how output can be produced

for visualization. The separate steps are almost identical for the spherical and realistic case, only the input related to the volume conductor model differs.

2.2.1 Example scripts for spherical models

An overview of the example scripts related to the spherical head representation is listed here:

Sa) **sphere_cg_tet_transfer.py**: CG-FEM with tetrahedral mesh and transfer matrix approach, partial integration/St. Venant/Subtraction/Whitney source models

Sb) **sphere_cg_tet_transfer.m**: CG-FEM with tetrahedral mesh and transfer matrix approach, partial integration/St. Venant/Subtraction/Whitney source models (MATLAB interface)

Sc) **sphere_cg_hex_direct.py**: CG-FEM with hexahedral mesh and direct approach, St. Venant source model

Sd) **sphere_dg_tet_transfer.py**: DG-FEM with tetrahedral mesh and transfer matrix approach, partial integration source model

Se) **sphere_udg_transfer.py**: UDG-FEM with the transfer matrix approach, partial integration source model

2.2.2 Example scripts for realistic models

The example scripts related to the realistic head models are listed here:

Ra) **realistic_cg_tet_transfer.py**: CG-FEM with tetrahedral mesh and transfer matrix approach, partial integration/St. Venant/Subtraction/Whitney source models

Rb) **realistic_dg_tet_direct.py**: DG-FEM with tetrahedral mesh and direct approach, partial integration source model

Rc) **realistic_udg_transfer.py**: UDG-FEM with transfer matrix approach, partial integration source model

2.3 A closer look: CG-FEM using a tetrahedral mesh, the transfer matrix approach and different source models (Python interface)

In this section, we will have a detailed look at each of the steps necessary to compute the EEG forward solution using the Python interface of DUNEuro. This refers to example scripts Sa) using a spherical head model and Ra) with a realistic model. In both cases, a tetrahedral volumetric mesh is used for the head volume conductor model and the EEG potential is computed using the standard Continuous Galerkin Finite Element Method (CG-FEM) for different

source models (Partial Integration, St. Venant, Subtraction and Whitney). The transfer matrix approach is used to speed up computations for the typical case that the number of dipoles exceeds the number of sensors. As all Python example scripts, they can be executed using the terminal command '`python3` *`filename`*`.py`'.

2.3.1 Step I)

In this preparatory step, we need to import the DUNEuro library using the following command:

```
import duneuropy as dp
```

Additionally, we require some other libraries such as `numpy` for handling arrays and libraries for visualization, which can be installed using *pip*.

2.3.2 Step II)

In this step, the path to the folder which contains the input data and the path to a folder where the output data should be stored need to be indicated. The input data consist of the tetrahedral mesh, the conductivity file, dipoles, electrodes and in the spherical case, the (quasi-)analytical solution that we will use for the comparison with the numerical result.

2.3.3 Step III)

Here, we create the driver with parameters related to our FEM approach and the volume conductor head model.

```
config = {
  'type' : 'fitted',
  'solver_type' : 'cg',
  'element_type' : 'tetrahedron',
  'volume_conductor' : {
    'grid.filename' : filename_grid,
    'tensors.filename' : filename_tensors
  }
}
driver = dp.MEEGDriver3d(config)
```

Since we want to use the classical CG-FEM approach, we specify the 'type' as 'fitted' and 'solver_type' as 'cg'. The volume conductor is specified as the mesh file and the conductivity file, which contains a list of conductivity values in the order of the labels within the mesh file. Note that all input files, including the mesh file, use dots as decimal separators.[1] Alternatively, the volume conductor can also be specified by directly passing the elements, nodes and labels of the mesh, as demonstrated in Sc) and described in more detail in 2.5.

[1] Numerical formatting differs across countries, which can lead to errors while reading the mesh in case a different decimal specifier (e.g., a comma) is expected due to system-specific settings. The terminal command `sudo update-locale LC_NUMERIC=C` can be used to change the numerical formatting default system-wide after restarting.

2.3.4 Step IV)

Next, the electrode positions are read from the text file whose path was defined earlier. Now, the electrode positions are passed to DUNEuro using the `setElectrodes` method. Here, we choose the closest subentity within our grid with codimension 3, which means that we move each electrode to the closest node within the given mesh. Note that this node could also be located within the head model.

```
config = {
  'type' : 'closest_subentity_center',
  'codims' : [3]
}
driver.setElectrodes(electrodes, config)
```

Another option is to project the electrode positions orthogonally to the head surface by setting `'type' : 'normal'`.

2.3.5 Step V)

In the next step, we use the transfer matrix approach to compute the EEG leadfield. This consists of several sub-steps. First, the transfer matrix is computed.

```
config = {
  'solver' : {
    'reduction' : 1e-10
  }
}
tm = driver.computeEEGTransferMatrix(config)
```

Optionally, parameter choices can be passed to the solver, in this case the relative reduction is set to 1e-10. This value should not be too large in order to ensure an adequate accuracy.

Next, the source model configuration is defined. Depending on the source model, there are different mandatory and optional parameters. For the partial integration source model, for instance, no parameters are needed. For the St. Venant source model, several parameters are necessary, e.g., if the so-called St. Venant condition should be fulfilled (`'restrict' = 'true'`). For a more detailed description of the source models, see [5, 6, 7, 8, 9, 10].

After reading the dipole locations and moments from our input file, we can pass them together with the source model configurations and apply the transfer matrix:

```
for sm in source_model_configs:
  lf = driver.applyEEGTransfer(tm_eeg, dipoles, {
    'source_model' : source_model_configs[sm],
    'post_process' : True,
    'subtract_mean' : True
  })
  solutions[sm] = np.array(lf[0])
```

The 'subtract_mean' parameter indicates that the resulting EEG leadfield will be average referenced. The setting 'post_process' : True, is relevant for the subtraction source model and indicates that the analytical portion of the solution is added to the numerically computed correction potential, see [6, 7].

2.3.6 Step VI)

Here, the mesh is written in .vtk format using the *write* function of DUNEuro. Additionally, the first dipole and the solution for this dipole using the St. Venant source model at the electrodes are produced as output. These three files can be visualized using ParaView [1], an open-source visualization toolbox, the result is shown in Fig 3. In order to reproduce Fig 3 (left subfigure), the attached ParaView state **sphere_cg_tet_transfer.psvm** can be loaded within Paraview via **File** → **Load State** which opens a dialog window in which the paths to the three output files of Sa) need to be indicated.

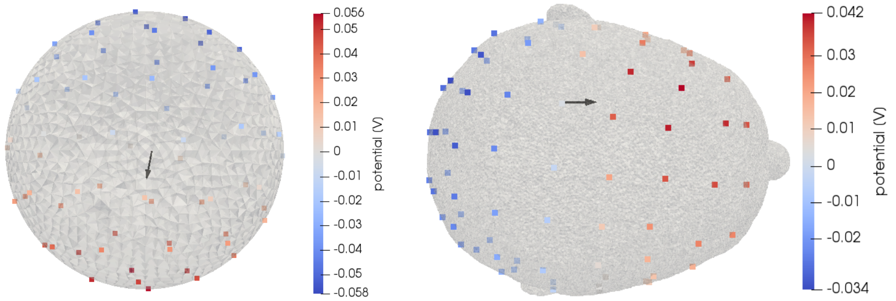

Figure 3. EEG forward solution at electrode positions computed using CG-FEM for a test dipole (magnified, shown as black arrow) in tetrahedral spherical model (left) and realistic model (right).

In the spherical case, we can also load the (quasi-)analytical solution and compare it to the numerical one. We compute the topography and magnitude errors (RDM and lnMAG) and visualize the errors for the test dipoles at different eccentricities, see Fig 4.

Additionally, we can call the function *print_citations* to get a list of relevant publications which provide further information on the software as well as the source models and finite element methods that were used.

2.4 How to use the MATLAB interface

The interface to MATLAB is very similar to the Python interface. Instead of passing parameters through Python dictionaries, MATLAB structure arrays are used. An example which computes the EEG leadfield using the same configurations as Sa) using the MATLAB interface is provided by Sb).

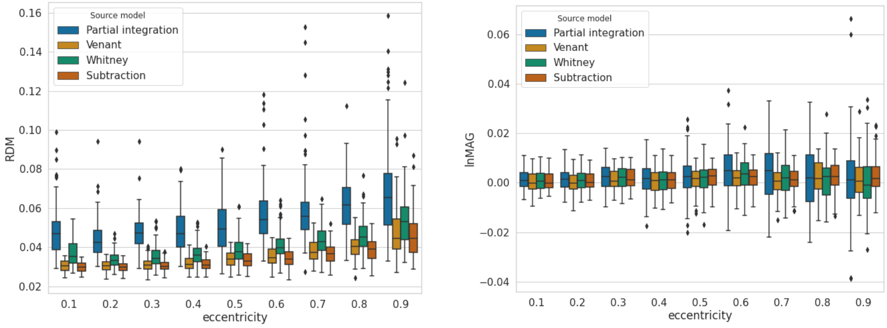

Figure 4. RDM (left) and lnMAG (right) errors for CG-FEM computation in tetrahedral spherical model using different source models.

2.5 How to use hexahedral meshes

Example script Sc) gives an example how hexahedral meshes can be passed instead of tetrahedral ones. The only difference is in Step III). When passing the volume conductor,

```
'element_type' : 'hexahedron'
```

is used instead of ‘tetrahedron‘.

In this example script, an alternative way to pass the volume conductor is shown instead of passing the mesh and tensors via input files, which is possible for both tetrahedral and hexahedral meshes. A list of node coordinates is passed in combination with a list of elements, containing the indices (starting at 0) of the nodes associated with each element. Additionally, a list of labels (starting at 0) which correspond to the list of conductivities is provided. Note that the mesh size of the regular hexahedral grid created in Sc) is 1 mm in order to achieve a reasonable resolution even for the narrow CSF tissue layer. This results in 3 342 701 nodes and 3 262 312 elements, leading to a large system of linear equations that needs to be solved when computing the EEG solution.

2.6 How to use the direct approach when computing the forward solution

In case the solution needs to be computed for a small set of dipoles, e.g., for visualization purposes, DUNEuro can compute the EEG leadfield directly without first computing the transfer matrix. In step V), instead of using the functions *computeEEGTransferMatrix* and *applyEEGTransfer* explained above, the function *solveEEGForward* is used. The EEG solution is computed and can then be directly written out, before it is evaluated at the electrode positions using the function *evaluateAtElectrodes*. Example script Sc) and Rb) show how to carry out these steps. The visualized output of these scripts is shown in Fig 5.

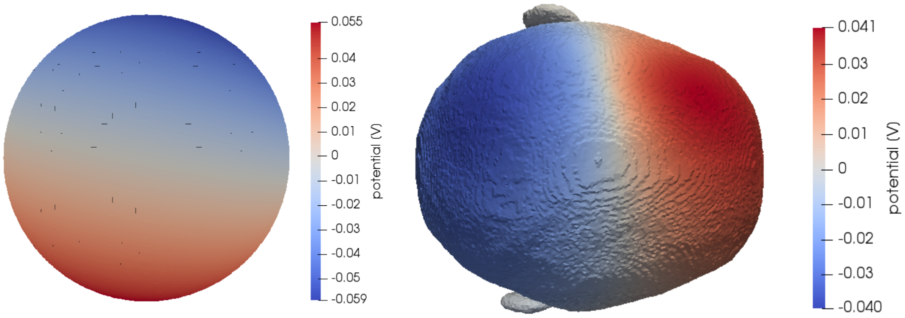

Figure 5. Visualization of EEG solution using the direct approach for spherical head model using CG-FEM (left) and for the tetrahedral realistic model using DG-FEM (right).

2.7 How to use other Finite Element Methods

In the detailed example in subsection 2.3, the standard (CG-)FEM is used for the EEG forward computation. However, DUNEuro offers additional discretization methods, the examples below will explain how they can be used.

2.7.1 DG-FEM

In general, the user can easily switch between different FEM discretizations by modifying the configuration when setting up the driver in Step III). Example scripts Sd) and Rb) show how to use DG-FEM for a spherical and realistic test case. As shown in the example files, the main difference is that the ‘solver_type‘ is changed to ‘dg‘. Additionally, the solver requires some parameters, such as the penalty parameter.

```
config = {
  'type' : 'fitted',
  'solver_type' : 'dg',
  'element_type' : 'tetrahedron',
  'volume_conductor' : {
    'grid.filename' : filename_grid,
    'tensors.filename' : filename_tensors
  },
  'solver' : {
    'edge_norm_type' : 'houston',
    'penalty' : 20,
    'reduction' : 1e-10,
    'scheme' : 'sipg',
    'weights' : 'tensorOnly',
  }
driver = dp.MEEGDriver3d(config)
```

For a more detailed description of the DG method, we refer for EEG to [11] and for MEG to [12]. The output of Rb) is depicted in Fig 5 (right subfigure).

2.7.2 UDG-FEM

Similarly, the code in Step III) can be modified for the unfitted discontinuous Galerkin method (UDG-FEM), as shown by example scripts Se) and Rc). In this case, we need to pass the different options for the FEM type (set 'type' to 'unfitted' and 'solver_type' to 'udg'), but also pass the volume conductor differently. For this unfitted method, a hexahedral coarse background mesh is used, which does not resolve the head's geometry. Instead, the boundary of each tissue type is represented by level set information.

For the spherical case, within the configuration for the driver creation, the volume conductor can be passed by specifying the characteristics of the background mesh, in this case, there are 30^3 cells within the bounding box $[30, 220]^3$ in mesh coordinates.

```
'volume_conductor' : {
  'grid' : {
  'cells' : (30, 30, 30),
  'upper_right' : (220, 220, 220),
  'lower_left' : (30, 30, 30),
  'refinements' : 0
  }
}
```

Since this coarse fundamental grid does not match with the actual head geometry, the tissue characteristics need to be specified in addition to that, as done here for the spherical case.

```
'domain' : {
  'domains' : ['skin', 'skull', 'csf', 'brain'],
  'level_sets' : ['outer_skin', 'skin_skull', 'skull_csf', '
                                 csf_brain'],
  'brain.positions' :  'iiii',
  'csf_brain.radius' : 78,
  'csf.positions' : 'iiie',
  'skull_csf.radius' : 80,
  'skull.positions' : 'iiee',
  'skin_skull.radius' : 86,
  'skin.positions' : 'ieee',
  'outer_skin.radius' : 92
```

A list of domain types is indicated, together with their respective position (internal/external of the surfaces). Also, all surfaces need to be listed in combination with their spherical characteristics, i.e., the radius (shown above) and the type and center, done within a for loop within the code.

```
'type' : 'sphere',
'center' : (127, 127, 127)
```

Additionally, the number of compartments (tissue types) needs to be indicated (four in the spherical case) and the solver takes additional parameters, similar to the DG case. Here, the conductivity values are also passed. The compartments of the source and the sensors need to be indicated as well in the source model and transfer matrix configurations, respectively.

For the realistic case, we need to specify `'type' : 'image'` instead of `'sphere'` and the actual level set information is passed using the `'data'` option. Here, we pass the npy arrays for each surface, which are 3-dimensional arrays which have zero points at the respective surface. Note that computations using the UDG method, in particular in the realistic mesh, is memory- and time-consuming. In order to successfully run script Rc), which involves approximately 6.5 million degrees of freedom, we recommend at least 32 GB of RAM and to use the TBB library for thread-parallelization.

For a more detailed description of the UDG method, we refer to [13, 10].

A visualization of the EEG leadfield for a test dipole using the above-mentioned example scripts for UDG-FEM is presented in Fig 6.

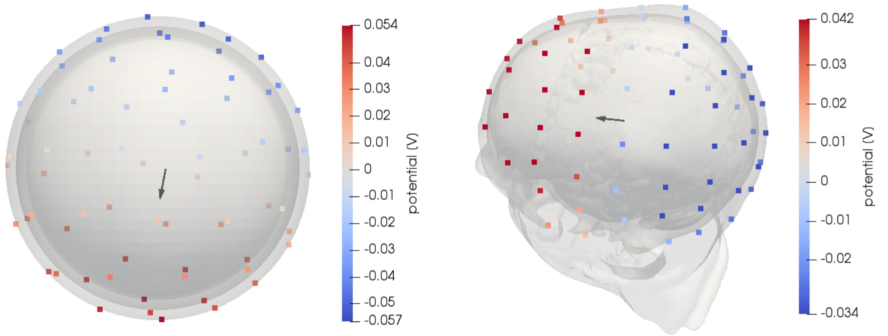

Figure 6. EEG forward solution at electrode positions computed using UDG-FEM for a test dipole (magnified, shown as black arrow) in unfitted spherical (left) and realistic model (right).

2.8 Outlook and further information

The example scripts presented show important features of DUNEuro related to the computation of the EEG forward solution using modern FEM discretizations. However, there are several additional features available which are not explained in detail here, such as the MEG forward solution computation which consists of the same main steps shown here [14, 12, 15], or geometry-adapted hexahedral meshes [10]. Moreover, we focus on the interface to the Python scripting language which is free and open-source. DUNEuro also offers bindings to MATLAB as mentioned in 2.4 and illustrated by example script Sb), and can also be used directly using C++ code. In the future, we are planning to extend the documentation of our software toolbox and provide further tutorials and example scripts on the DUNEuro homepage (`http://www.duneuro.org`) and on the GitLab repository (`https://gitlab.dune-project.org/duneuro`) which also offers a platform for issue tracking, bug reporting, and discussions.

References

[1] Ahrens J, Geveci B, Law C. ParaView: An End-User Tool for Large Data Visualization, Visualization Handbook. Elsevier; 2005.

[2] Geuzaine C, Remacle JF. Gmsh: A 3-D finite element mesh generator with built-in pre- and post-processing facilities. International Journal for Numerical Methods in Engineering. 2009;79(11):1309–1331.

[3] De Munck J, Peters MJ. A fast method to compute the potential in the multisphere model. IEEE Trans Biomed Eng. 1993;40(11):1166–1174.

[4] Piastra MC, Schrader S, Nüßing A, Antonakakis M, Medani T, Wollbrink A, et al.. The WWU DUNEuro reference data set for combined EEG/MEG source analysis; 2020. Available from: `https://zenodo.org/record/3888381`.

[5] Bauer M, Pursiainen S, Vorwerk J, Köstler H, Wolters CH. Comparison study for Whitney (Raviart–Thomas)-type source models in finite-element-method-based EEG forward modeling. IEEE Transactions on Biomedical Engineering. 2015;62(11):2648–2656.

[6] Drechsler F, Wolters CH, Dierkes T, Si H, Grasedyck L. A Full Subtraction Approach for Finite Element Method Based Source Analysis Using Constrained Delaunay Tetrahedralisation. NeuroImage. 2009;46(4):1055–1065. doi:10.1016/j.neuroimage.2009.02.024.

[7] Wolters C, Köstler H, Möller C, Härdtlein J, Grasedyck L, Hackbusch W. Numerical Mathematics of the Subtraction Method for the Modeling of a Current Dipole in EEG Source Reconstruction Using Finite Element Head Models. SIAM Journal on Scientific Computing. 2007;30(1):24–45. doi:10.1137/060659053.

[8] Miinalainen T, Rezaei A, Us D, Nüßing A, Engwer C, Wolters CH, et al. A realistic, accurate and fast source modeling approach for the EEG forward problem. NeuroImage. 2019;184:56–67.

[9] Vorwerk J, Cho JH, Rampp S, Hamer H, Knösche TR, Wolters CH. A guideline for head volume conductor modeling in EEG and MEG. NeuroImage. 2014;100:590–607.

[10] Nüßing A. Fitted and Unfitted Finite Element Methods for Solving the EEG Forward Problem [Ph.D. thesis]. Westfälische Wilhelms-Universität Münster; 2018. Available from: `http://nbn-resolving.de/urn:nbn:de:hbz:6-67139436770`.

[11] Engwer C, Vorwerk J, Ludewig J, Wolters CH. A Discontinuous Galerkin Method to Solve the EEG Forward Problem Using the Subtraction Approach. SIAM Journal on Scientific Computing. 2017;39(1):B138–B164. doi:10.1137/15M1048392.

[12] Piastra MC, Nüßing A, Vorwerk J, Bornfleth H, Oostenveld R, Engwer C, et al. The Discontinuous Galerkin Finite Element Method for Solving the MEG and the Combined MEG/EEG Forward Problem. Frontiers in Neuroscience. 2018;12:30.

[13] Nüßing A, Wolters CH, Brinck H, Engwer C. The Unfitted Discontinuous Galerkin Method for Solving the EEG Forward Problem. IEEE Transactions on Biomedical Engineering. 2016;63(12):2564–2575. doi:10.1109/TBME.2016.2590740.

[14] Piastra MC, Nüßing A, Vorwerk J, Clerc M, Engwer C, Wolters CH. A Comprehensive Study on EEG and MEG Sensitivity to Cortical and Subcortical Sources. Human Brain Mapping. 2020; p. Forthcoming.

[15] Piastra MC. New finite element methods for solving the MEG and the combined MEG/EEG forward problem [Ph.D. thesis]. Westfälische Wilhelms-Universität Münster; 2019. Available from: http://nbn-resolving.de/urn:nbn:de:hbz:6-53199662090.
